# Thermochemical and photochemical aerobic oxidation of benzylic alcohols in the presence of Cu(ii) nitrate, DDQ, and their combination

**DOI:** 10.1039/d5ra08493h

**Published:** 2026-03-19

**Authors:** Hamzeh Veisi, Amin Rostami, Kamal Amani, Atefeh Charabeh

**Affiliations:** a Department of Chemistry, Faculty of Science, University of Kurdistan Sanandaj Iran a.rostami@uok.ac.ir; b Department of Chemistry, Sanandaj Branch, Islamic Azad University P.O. Box 618 Sanandaj Iran

## Abstract

The selective oxidation of benzylic alcohols to carbonyl compounds under mild conditions remains a significant challenge in synthetic chemistry. Here, we report four cost-effective and efficient strategies for the selective aerobic oxidation of benzylic alcohols to carbonyl compounds in the presence of Cu(ii) nitrate, DDQ, and their combination under thermochemical and photochemical conditions in CH_3_CN as a solvent: (1) thermally-assisted DDQ organocatalysis at 60 °C, (2) DDQ photochemical catalysis under light irradiation, (3) Cu(NO_3_)_2_·3H_2_O/DDQ catalyst system, and (4) light-driven (blue LEDs 9 W) Cu(NO_3_)_2_·3H_2_O/DDQ photocatalytic system. Among these methods, the photoactive DDQ/Cu(NO_3_)_2_·3H_2_O catalytic system demonstrated the highest performance. These methods offer several notable advantages, including the use of oxygen as the terminal oxidant and the utilization of commercially available, inexpensive catalysts, making the process both economical and environmentally friendly. Furthermore, they produce environmentally benign water as the sole byproduct, offer 60–98% product yields, and allow for straightforward isolation and purification. The combination of these features makes these protocols both practical and sustainable for the selective oxidation of benzylic alcohols to carbonyl compounds under ambient aerobic conditions.

## Abstract 2

The aerobic oxidation of benzyl alcohols in the presence of DDQ and Cu(NO_3_)_2_·3H_2_O/DDQ catalyst system is described.

## Graphical abstract

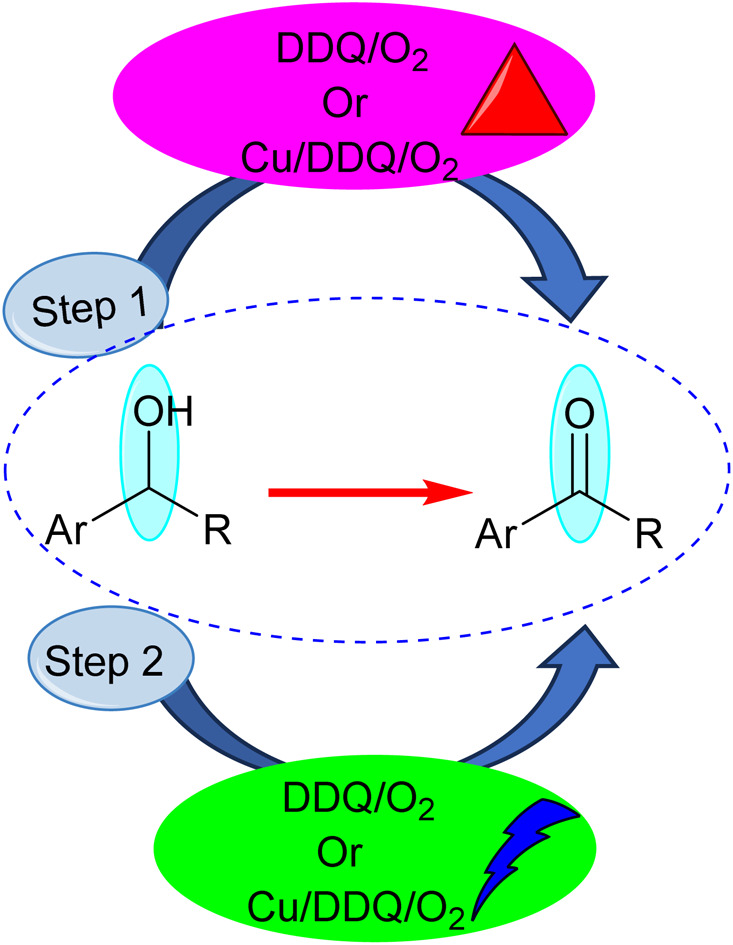

## 1. Introduction

Chemical structures that contain carbonyl groups, including aldehydes and ketones, are commonly found and highly desired frameworks because of their broad uses in pharmaceuticals and agrochemicals for research on fine chemicals and the preparation of functional materials.^1^ Converting alcohols to carbonyl compounds through oxidation is extremely important in both the laboratory and chemical industry.^2^ Traditionally, these changes have been made using stoichiometric inorganic chemicals like NaIO_4_,^3^ pyridinium chlorochromate (PCC), Hydrated ruthenium dioxide (RuO_2_·*x*H_2_O),^4^ DDQ/(MnOAc)_3_,^5^ N-doped graphene,^6^ manganese(iv),^7^*etc.* These techniques are linked to harsh reaction conditions and potentially low atom efficiency. Therefore, there continue to be obstacles in the selective oxidation of benzylic alcohols.^8^ New developments in catalytic studies have resulted in the utilization of eco-friendly oxidizing agents like molecular oxygen and hydrogen peroxide, along with efficient and selective reusable catalysts made from transition metals such as Pt, Pd, Cu, Ru, Ir, Rh, Fe, Au, and others.^9^ Utilizing molecular oxygen as a green oxidant in oxidative methods is a more sustainable choice, similar to oxidase catalytic reactions.^10^ In this context, the advancement of catalytic methods utilizing affordable and environmentally friendly molecular oxygen as the oxidizing agent has been notable.^4,11^ The introduction of cost-effective and eco-friendly catalytic systems for the aerobic oxidation of alcohols represents a critical research objective. Despite significant progress in copper-catalyzed aerobic oxidation, Cu-based systems often suffer from limitations such as the need for expensive ligands, sensitivity to air and moisture, narrow substrate scope, and, in some cases, prolonged reaction times or low selectivity for benzylic alcohols.^12^ These drawbacks hinder their practical application in large-scale and sustainable synthesis.

Copper salts are frequently employed in organic synthesis for their roles as oxidants, mediators, and catalysts. Copper nitrate is a well-known cupric salt that appears as a blue crystalline solid, known for its low toxicity, affordability, easy availability, and operational simplicity.^13^ Copper nitrate exists in five hydrates, with the trihydrate variety being the most prevalent.^14^ There has been a significant advancement in organometallic chemistry, with copper nitrate proving to be a useful catalyst or promoter.^15^ Because of its distinct physical characteristics and varying reactivity, as well as its environmentally friendly qualities, copper nitrate has become increasingly popular among organic chemists and is now seen as a versatile reagent for organic synthesis.^14*a*,16^ In recent years, photocatalytic oxidation of alcohols has emerged as a promising green alternative to traditional methods. For instance, a recent study demonstrated the photocatalytic oxidation of biomass-derived alcohols using a novel heterojunction catalyst, achieving excellent yields under mild conditions.^17^ Similarly, another work reported the photoelectro-catalytic oxidation of alcohols to carbonyl compounds, highlighting the potential of light-driven strategies for sustainable synthesis.^18^ Despite these advances, challenges such as limited substrate scope, the need for specialized photocatalysts, and scalability issues remain to be addressed.

In synthetic organic chemistry, 2,3-dichloro-5,6-dicyano-*p*-benzoquinone (DDQ) is recognized as a potent oxidation reagent.^19^ Having stoichiometric oxidant properties, it has facilitated various reactions, such as alcohol oxidation,^20^ condensations,^21^ aromatization,^22^ oxidative cycloadditions,^23^ water splitting,^24^ and more. Adding more than a stoichiometric amount and generating 2,3-dichloro-5,6-dicyano-hydroquinone (DDQH_2_) as a side reaction makes it costly and challenging to purify from the excess DDQ, thus impacting both cost and atom economy. Researchers have investigated using a catalytic amount of DDQ and a co-oxidant to address these deficiencies in organic synthesis.^25^ Hu *et al.* presented the TBN/O_2_ co-oxidant system, which has since found extensive application in DDQ catalytic reactions.^26^ NO, produced by TBN, can trigger the activation of molecular oxygen and transform into NO_2_, which acts as a co-oxidant. Therefore, the DDQ/TBN/O_2_ system can facilitate cost-effective and atom-efficient oxidative reactions. The ground-state DDQ has an oxidation potential of 0.51 V (*versus* SCE), and visible light-activated DDQ exhibits powerful oxidizing capabilities.^27^ Fukuzumi, Ohkubo, and colleagues discovered that exposing excited state DDQ to blue light increases its single-electron transfer (SET) oxidation potential from 0.51 V to 3.18 V (*vs.* SCE) through triplet excitation (^3^DDQ*) or 3.80 V (*vs.* SCE) through singlet excitation (^1^DDQ*).^28^ This approach represents a fundamental advancement in photoactivated DDQ catalysis, enabling diverse transformations including C–C bond formation, α-activation of O/S-containing compounds, benzylic oxygenation, and direct aromatic ring functionalization.^29^

Organocatalysts are powerful tools in synthetic chemistry, offering metal-free, environmentally friendly, and mechanistically diverse pathways for complex transformations. Bifunctional organocatalysts enable highly enantioselective C–C bond-forming reactions, underscoring the versatility of organocatalysis beyond traditional oxidation chemistry.^30^ DDQ has been extensively reviewed as an organic oxidant, demonstrating broad applications in hydride-transfer and single-electron-transfer processes for the oxidation of benzylic alcohols, ethers, and related substrates.^25*c*^ Despite these advances, most organocatalytic systems either focus on asymmetric induction or require stoichiometric DDQ with strong co-oxidants. In continuation of the catalytic application of laccase/DDQ^31^ and copper(ii) nitrate^32^ This study focused on cost-effective oxidizing agents for the aerobic oxidation of benzylic alcohols to the corresponding aldehydes and ketones. Although DDQ has been widely used as an organocatalyst in oxidative transformations, its application in aerobic oxidation of benzylic alcohols has remained limited to systems requiring mediators, harsh conditions, or stoichiometric quantities. In contrast, the present study introduces four previously unreported catalytic strategies: (a) mediator-free DDQ under thermal conditions, (b) Cu(NO_3_)_2_·3H_2_O/DDQ under thermal conditions, (c) mediator-free DDQ under blue LED irradiation, and (d) Cu(NO_3_)_2_·3H_2_O/DDQ under blue LED irradiation. These protocols operate under exceptionally mild aerobic conditions, significantly reduce DDQ consumption, eliminate the need for external oxidants other than O_2_, and enable room-temperature photocatalytic oxidation. To the best of our knowledge, no prior report has described the aerobic oxidation of benzylic alcohols using these mediator-free or Cu-assisted DDQ-catalytic systems.

## 2. Results and discussions

Initially, aerobic oxidation of 1-phenylethanol was selected as the model reaction to assess the performance of the DDQ organocatalyst (Table 1). The effects of catalyst quantity and temperature were systematically studied to determine the optimal conditions. As illustrated in Table 1, the use of 25 mol% DDQ at 60 °C was identified to be the optimal reaction condition (Table 1, entry 4). Subsequently, to enhance both cost-effectiveness and environmental sustainability, copper(ii) nitrate was incorporated as a co-catalyst in combination with a reduced amount of DDQ (Table 1, entries 7–9). The best result was obtained, with 10 mol% copper(ii) nitrate in combination with 15 mol% DDQ (Table 1, entry 8). To evaluate the catalytic effect, the reaction was examined in the absence of the copper(ii) nitrate trihydrate/DDQ catalytic system. Notably, no progress of response was observed even after 24 h (Table 1, entry 10). Fortunately, when air (atmospheric oxygen) was used instead of pure molecular oxygen, the reaction proceeded smoothly, albeit with a slightly longer reaction time (Table 2, entry 11). To investigate the role of oxygen as the primary oxidant, the reaction was conducted under an N_2_ atmosphere. After 24 h, the product yield reached only 20% (Table 1, entry 12).

**Table 1:** Optimizing the aerobic oxidation reaction conditions for 1-phenylethanol with DDQ as an organocatalyst^a^

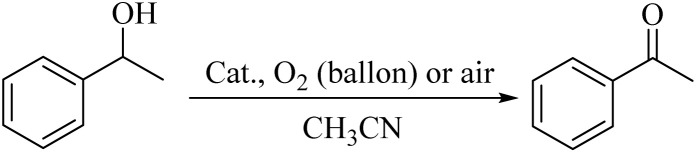						
Entry	DDQ (mol%)	Cu(NO_3_)_2_·3H_2_O (mol%)	O_2_ or air	*T* (°C)	Time (h)	GC yield (%)
1	30	—	O_2_	70	9	87
2	30	—	O_2_	60	10	85
3	30	—	O_2_	40	24	45
4	25	—	O_2_	60	10	90
5	15	—	O_2_	60	24	70
6	5	—	O_2_	60	24	38
7	20	10	O_2_	60	6	93
8	15	10	O_2_	60	8	93
9	10	10	O_2_	60	20	77
10	—	—	—	60	24	0
11	15	10	Air	60	22	86
12	15	10	N_2_	60	24	20

a Conditions: substrate (1 mmol), catalyst (Cu(NO_3_)_2_·3H_2_O and DDQ) in CH_3_CN solvent (2 mL).

**Table 2:** Aerobic oxidation of benzylic alcohols in the presence of DDQ or Cu(NO_3_)_2_·3H_2_O/DDQ catalysts

Entry	Substrate	DDQ^a^		Cu(NO_3_)_2_·3H_2_O/DDQ^b^		*M*_p_. (°C) (DNPH)
		*t* (h)	Isol. Yield (%)	*t* (h)	Isol. Yield (%)	
1	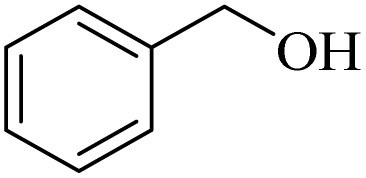	11	84	8	90	239–241
2	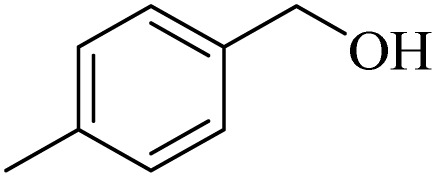	8	85	6	91	231–233
3	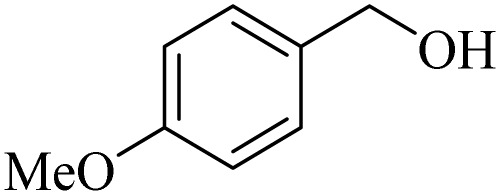	1	90	0.5	93	195–197
4	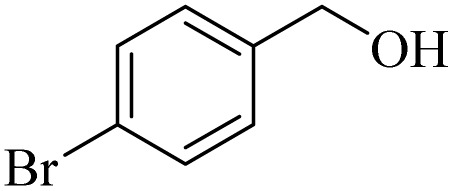	11	81	9	87	262–264
5	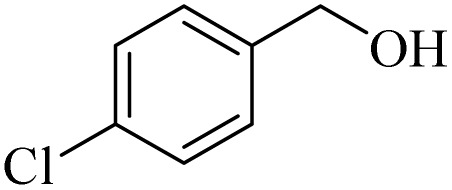	12	80	10	87	262–265
6	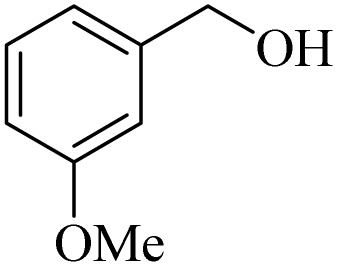	24	77	24	79	227–229
7	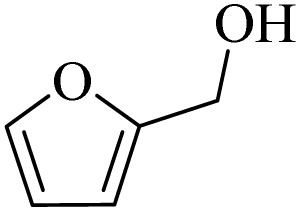	24	60	24	60	150–152
8	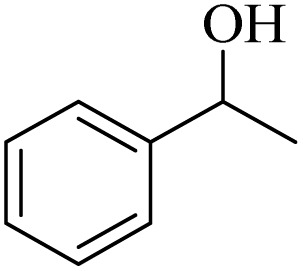	10	88	8	90	245–248
9	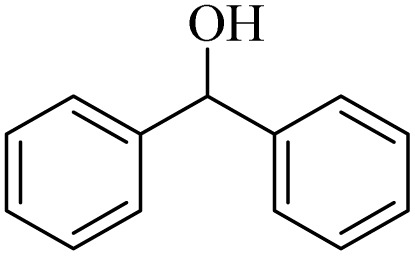	7	90	6	93	140–143
10	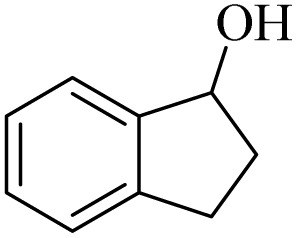	9	88	7	90	256–258

a Conditions: substrate (1 mmol) and DDQ (25 mol%) in MeCN (2 mL) under O_2_ (balloon) at 60 °C.

b Conditions: substrate (1 mmol) and Cu(NO_3_)_2_·3H_2_O (10 mol%)/DDQ (15 mol%) in MeCN (2 mL) under O_2_ (balloon) at 60 °C temperature.

After optimization, both catalytic protocols DDQ/O_2_ and DDQ/Cu(ii)/O_2_ were evaluated for aerobic oxidation of various types of benzylic alcohols under the best reaction conditions (Table 1, entries 4 and 8) to produce the corresponding products, as shown in Table 2. It is important to note that primary benzylic alcohols underwent selective oxidation to produce the corresponding benzaldehyde derivatives, without over-oxidation to benzoic acid. Benzyl alcohols with electron-donating groups showed quicker reaction times than those with electron-withdrawing groups. This oxidation protocol resulted in a 60% efficiency in oxidizing the alcohol group of furan-2-ylmethanol (Table 2, entry 7). Also, the effectiveness of both catalytic protocols (DDQ/O_2_/heat and Cu/DDQ/O_2_/heat) was evaluated for secondary benzylic alcohols, yielding highly satisfactory results (Table 2, entries 8–10).

Next, to develop a more energy-efficient protocol, we replaced thermal activation (heat) with visible-light irradiation (blue LEDs) in the DDQ photo-organocatalyst aerobic oxidation system. This photochemical approach eliminates the need for heating and maintains high reaction efficiency under milder conditions. Various experimental parameters were investigated to determine the optimal conditions (Table 3). Initially, the effect of DDQ catalyst amount on the model reaction was investigated (Table 3, entries 1–4). The optimal performance was achieved with 20 mol% DDQ (Table 3, entry 2). Then, a photochemical catalytic system comprising copper(ii) nitrate trihydrate and DDQ was employed to reduce the amount of DDQ. To establish optimal conditions, the model reaction was evaluated with varying concentrations of both copper(ii) nitrate trihydrate and DDQ (Table 3, entries 4–6). The reaction was performed in the absence of both DDQ and copper(ii) nitrate trihydrate. As evidenced by the results, no conversion was observed even after 24 h (Table 3, entry 7). The reaction was subsequently performed under open-flask conditions (using ambient air instead of pure molecular oxygen), affording the product in 85% yield after 12 h (Table 3, entry 8).

**Table 3:** Optimizing the aerobic oxidation reaction conditions for 1-phenylethanol using DDQ/blue LEDs as a photocatalyst^a^

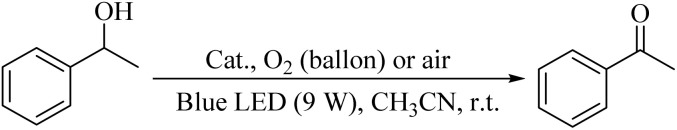					
Entry	DDQ (mol%)	Cu(NO_3_)_2_·3H_2_O (mol%)	O_2_ (balloon) or air	Time (h)	GC yield (%)
1	10	—	O_2_	24	68
2	20	—	O_2_	5	92
3	25	—	O_2_	5	92
4	5	5	O_2_	24	70
5	5	10	O_2_	0.5	97
6	10	10	O_2_	0.5	97
7	—	—	O_2_	24	0
8	5	10	Air	12	85

a Conditions: substrate (1 mmol), different amounts of DDQ/blue LEDs in CH_3_CN solvent (2 mL) under O_2_ (balloon) at room temperature.

Following the optimization of reaction conditions, various alcohol derivatives were studied in the presence of both DDQ/blue LEDs and Cu(NO_3_)_2_·3H_2_O/DDQ/blue LEDs photocatalyst systems (Table 4).

**Table 4:** Aerobic oxidation of alcohols in the presence of DDQ/blue LEDs and Cu(NO_3_)_2_·3H_2_O/DDQ/blue LEDs

Entry	Substrate	DDQ/blue LEDs^a^		Cu(NO_3_)_2_·3H_2_O/DDQ/blue LEDs^b^	
		*t* (h)	Isol. Yield (%)	*t* (h)	Isol. Yield (%)
1	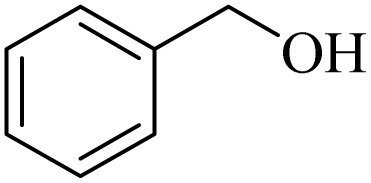	2	85	2	92
2	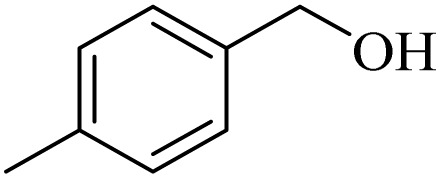	0.5	86	0.5	95
3	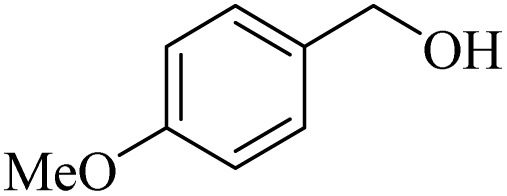	0.5	90	0.5	98
4	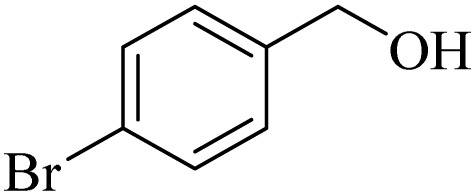	3	81	2	90
5	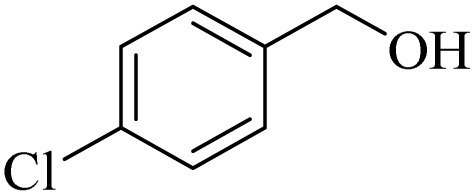	3	80	2	88
6	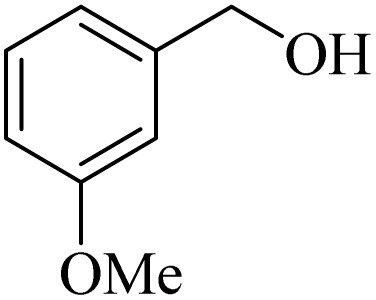	24	85	24	88
7	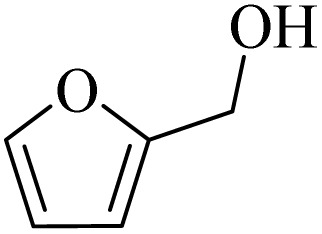	24	60	24	65
8	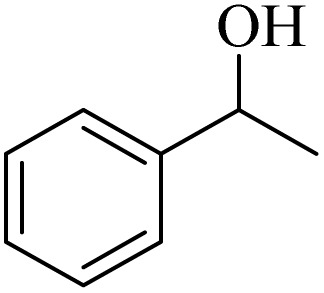	0.5	89	0.5	94
9	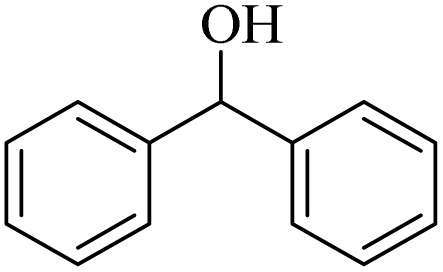	0.5	87	0.5	95
10	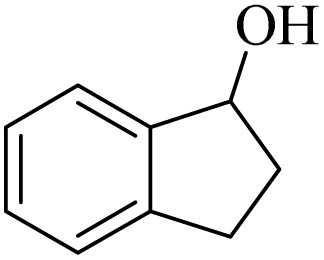	2	86	1	91

a Conditions: substrate (1 mmol) and DDQ (20 mol%)/blue LEDs (9 W) in MeCN (2 mL) under O_2_ (balloon) at room temperature.

b Conditions: substrate (1 mmol), Cu(NO_3_)_2_·3H_2_O (10 mol%), and DDQ (5 mol%)/blue LEDs (9 W) in MeCN (2 mL) under O_2_ (balloon) at room temperature.

As a result, the photocatalyst system, which consists of Cu(NO_3_)_2_·3H_2_O (10 mol%) and DDQ (5 mol%), offers the advantage of a shorter reaction time, reduced DDQ consumption, and mild reaction conditions.

**Scheme 1:**
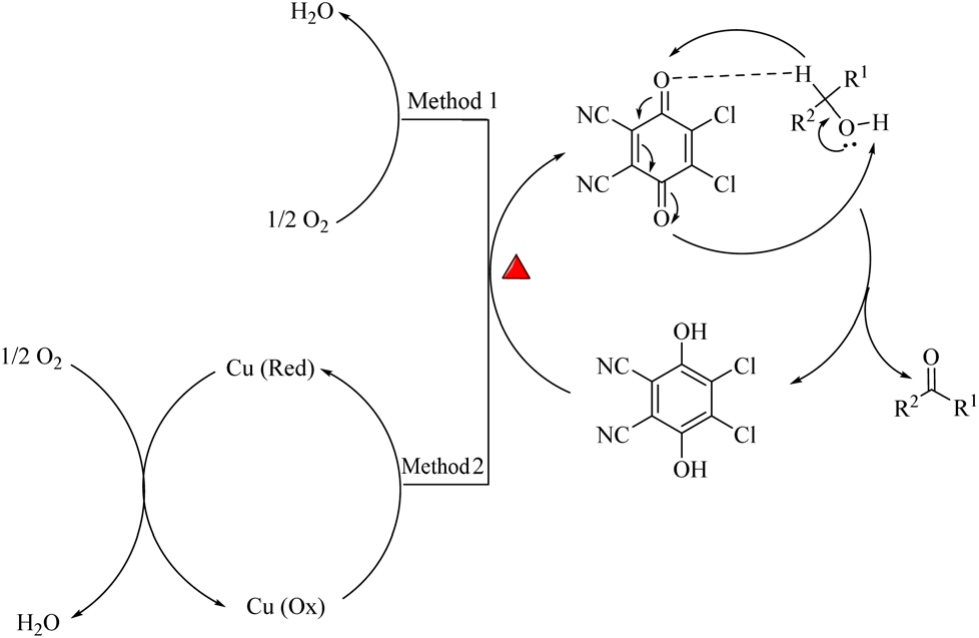
The proposed mechanism for the aerobic oxidation of alcohols in the presence of the DDQ and Cu/DDQ catalysts.

Although the mechanism of aerobic oxidation of benzylic alcohols to aldehydes and ketones by DDQ and Cu/DDQ is not precisely determined, based on previous studies, a plausible reaction mechanism is proposed in Scheme 1. It is postulated that the alcohol, in the presence of DDQ as a hydride ion acceptor, transfers a hydride ion to DDQ, thereby converting DDQ into DDQH. Concurrently, DDQH abstracts a proton from the hydroxyl group of the alcohol, yielding DDQH_2_ and the final product. DDQH_2_ is reoxidized to DDQ by molecular oxygen at 60 °C, completing the catalytic cycle (Method 1).^25*a*^ But in the presence of copper, Cu (O_*x*_) converts DDQH_2_ to DDQ, leading to the formation of DDQ and a reduced Cu. Ultimately, oxygen can regenerate the Cu (O_*x*_), thereby completing the catalytic cycle (Method 2).^25*b*,31*a*,33^

**Scheme 2:**
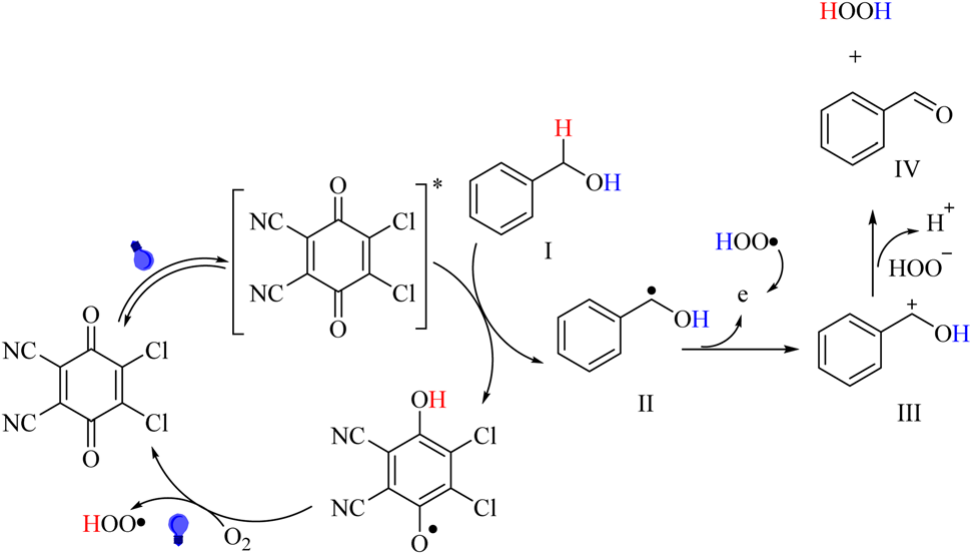
The proposed mechanism for the aerobic oxidation of alcohols in the presence of the DDQ/blue LEDs photo-organocatalyst.

The photo-organocatalyst mechanism is still unclear; however, a possible mechanism for the DDQ/blue LEDs-assisted oxidation of benzyl alcohols is outlined in Scheme 2, as per the literature. When exposed to visible light, the initial neutral form of DDQ transitions to its excited state, DDQ*.^8^ The DDQ* seizes hydrogen from benzyl alcohol (I) to produce the benzyl radical (II) and DDQ-H *via* the hydrogen atom transfer (HAT) mechanism. Afterward, DDQ-H transforms to DDQ while the oxygen in the ground state is simultaneously converted to a hydroperoxyl radical (HOO˙). The radical II gives away one electron, resulting in the formation of intermediate III. Following that, intermediate III removes a proton to yield the ultimate product IV.^8,25*b*,34^

**Scheme 3:**
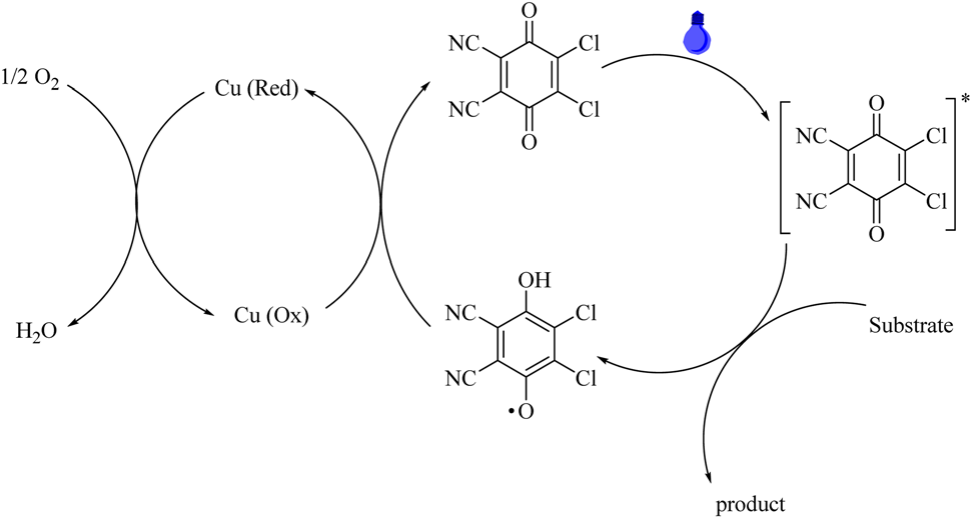
The proposed mechanism for the aerobic oxidation of alcohols in the presence of the Cu/DDQ/blue LEDs organocatalyst.

Also, although the precise process of Cu/DDQ/light photocatalyst mechanism remains uncertain, as per the literature, a plausible mechanism for the Cu/DDQ/light-assisted oxidation of benzyl alcohols is described in Scheme 3.^29*c*,35^ When exposed to visible light, DDQ transforms from its ground state to its excited state, DDQ*. Then the alcohol is oxidized to the product by DDQ*, and the side product DDQH_2_ is produced. Finally, DDQH_2_ is converted to DDQ using copper, and O_2_ can regenerate the Cu (O_*x*_), thereby completing the catalytic cycle, as shown in Scheme 3.^8,25*b*,31*a*,35,36^

Comparative evaluation of the present photocatalytic system's performance in 1-phenylethanol oxidation against previously reported methodologies (Table 5). As demonstrated in Table 5, copper nitrate/DDQ offers a cost-effective^31*c*,37^ and commercially available^37*b*^ photocatalyst system for the aerobic oxidation of 1-phenylethanol under visible light, achieving high yields at room temperature.

**Table 5:** Comparison of copper(ii) nitrate trihydrate/DDQ/blue LEDs, with other reagents or catalysts for oxidation of 1-phenylethanol (1 mmol)

Entry	Reaction conditions	*T* (°C)	Time (h)	Yield (%)	Ref.
1	CuCl·phen (2 eqs)/PhH/K_2_CO_3_ (2 eqs)/O_2_	Reflux	4	93	38
2	VO@g-C_3_N_4_ (10 mg)/H_2_O_2_ (1.5 mmol)/CH_3_CN (2 mL)/40 W domestic bulb	R.T.	1.5	97	39
3	CTF-Th@SBA-15 (100 mg)/benzotrifluoride (15 mL)/O_2_/blue light	R.T.	4	99	40
4	DDQ (20 mol%)/HNO_3_ (40 mol%)/DCM (0.2 M)/O_2_	R.T.	18	42	41
5	Cat [Cu^II^ (L^1^)] (3 mol%)/HFIP (5 mL)/O_2_ (1 atm)	R.T.	40	91.3	42
6	Cu(NO_3_)_2_·3H_2_O (10 mol%)/DDQ (5 mol%)/blue LEDs/O_2_/acetonitrile	R.T.	0.5	94	This work

## 3. Experimental

### 3.1. General procedure for the aerobic oxidation of alcohols by the DDQ organocatalyst

The alcohol (1.0 mmol), DDQ (0.25 mmol), and CH_3_CN (2 mL) were mixed using a flask equipped with a magnetic stirrer. An O_2_ balloon was used to fill the reaction tube with oxygen, resulting in an oxygen-rich environment. Afterward, the reaction mixture was vigorously agitated at 60 °C (for the duration specified in Table 2). The progress of the reaction was monitored by TLC (*n*-hexane/ethyl acetate, 4 : 1). Then, 5 mL of 10% NaOH was added to the reaction mixture. The product was then extracted with diethyl ether (3 × 10 mL), and the organic layer was dried over anhydrous sodium sulfate and concentrated under reduced pressure. In cases where the reaction did not proceed to completion, purification of the crude material was achieved by column chromatography on silica gel using a 3 : 1 mixture of *n*-hexane and ethyl acetate. The structures of the synthesized compounds were confirmed by conversion to their corresponding 2,4-dinitrophenylhydrazone (2,4-DNPH) derivatives and comparison of their melting points with literature values.^43^

### 3.2. General procedure for the aerobic oxidation of alcohols by Cu(NO_3_)_2_·3H_2_O/DDQ catalyst system

In a flask equipped with a magnetic stirrer, a mixture was prepared by combining 1.0 mmol of alcohol, 0.1 mmol of Cu(NO_3_)_2_·3H_2_O, 0.15 mmol of DDQ, and 2 mL of acetonitrile (CH_3_CN). The reaction mixture was vigorously agitated under an O_2_ atmosphere at 60 °C for the time indicated in Table 2. The progress of the reaction was tracked using TLC (*n*-hexane/ethyl acetate, 4 : 1). Then, 5 mL of 10% NaOH was added to the reaction mixture. The product was subsequently extracted using diethyl ether (3 × 10 mL), and the organic layer was dried with anhydrous sodium sulfate and concentrated under reduced pressure. In cases where the reaction did not proceed to completion, the crude material was purified using column chromatography on silica gel, utilizing a 3 : 1 combination of *n*-hexane and ethyl acetate.

### 3.3. General procedure for the aerobic oxidation of alcohols by DDQ/blue LEDs as a photo-organocatalyst

The flask with a magnetic stirrer was used to mix the alcohol (1.0 mmol), DDQ (0.2 mmol), and CH_3_CN (2 mL). Afterward, the reaction mixture was subjected to blue LED irradiation and vigorously agitated at room temperature under an oxygen atmosphere (balloon) for the specified duration listed in Table 4. The progress of the reaction was observed using TLC (*n*-hexane/ethyl acetate, 4 : 1). Then, 5 mL of a 10% NaOH solution was incorporated into the reaction mixture. The product was subsequently extracted using diethyl ether (3 × 10 mL), followed by drying the organic layer with Na_2_SO_4_ and concentrating it under reduced pressure. When the reaction did not reach completion, the crude material was purified using column chromatography on silica gel, utilizing a 3 : 1 ratio of *n*-hexane to ethyl acetate.

### 3.4. General procedure for the aerobic oxidation of alcohols using Cu(NO_3_)_2_·3H_2_O/DDQ/blue LEDs photocatalyst system

The flask, containing a magnetic stirrer, was used to combine the alcohol (1.0 mmol), Cu(NO_3_)_2_·3H_2_O (0.1 mmol), DDQ (0.05 mmol), and CH_3_CN (2 mL). To monitor the course of the reaction, TLC was performed using a mixture of *n*-hexane and ethyl acetate (4 : 1). Next, 5 mL of 10% sodium hydroxide solution was added. The desired compound was then obtained through repeated extraction with diethyl ether (3 × 10 mL). After drying the organic phase over sodium sulfate, it was concentrated under reduced pressure. In cases of incomplete reaction, purification was carried out *via* silica gel column chromatography using a 3 : 1 *n*-hexane to ethyl acetate mixture.

## 4. Conclusion

In summary, we have described four simple and economical catalytic procedures that use Cu(NO_3_)_2_·3H_2_O/DDQ/light, DDQ/light, Cu(NO_3_)_2_·3H_2_O/DDQ/heat, and DDQ/heat as the catalysts to oxidize alcohols into corresponding aldehydes/ketones with excellent conversion and selectivity with molecular oxygen. The thermal catalytic system (Cu(NO_3_)_2_·3H_2_O/DDQ) required heating at 60 °C in MeCN; the developed photocatalytic protocol successfully achieves alcohol oxidation at room temperature, offering a more energy-efficient and sustainable alternative. In the presence of Cu/DDQ/light (Cu(NO_3_)_2_·3H_2_O) (10 mol%) with DDQ (5 mol%) as the best method, the result shows that primary alcohols can be converted into the respective aldehydes in yields of 65–98%. Additionally, this method can produce ketones by oxidizing secondary alcohols in yields of 81–94%. The key advantages of the Cu(NO_3_)_2_·3H_2_O/DDQ photocatalytic system over the other three methods include significantly reduced DDQ consumption, higher product yields, and faster reaction rates at room temperature. Thus, the Cu(NO_3_)_2_·3H_2_O/DDQ and mediator-free DDQ oxidation of alcohols provide a significant incentive for developing aerobic oxidation protocols, particularly photocatalytic aerobic oxidation.

## Conflicts of interest

There are no conflicts to declare.

## Data Availability

The datasets used and analyzed during the current study are available from the corresponding author upon reasonable request.
